# Community Benefit: Policies, Practices, and Opportunities at the Half-Century Mark

**DOI:** 10.3389/fpubh.2020.00289

**Published:** 2020-07-27

**Authors:** Kevin Barnett

**Affiliations:** Center to Advance Community Health and Equity, Public Health Institute, Oakland, CA, United States

**Keywords:** community benefit, state and national policy, municipal property tax, social determinants of health, risk-based reimbursement

## Abstract

In the 50 years since the expansion of the legal definition of charity for tax-exempt hospitals, there have been periodic regulatory actions at the municipal, state, and federal level to quantify charitable contributions and justify the deferral of tax revenues. The movement toward risk-based reimbursement in the last decade creates an incentive for a shift in hospital leadership understanding and approach to community benefit programs and services. The historical interpretation of community benefit as an issue of compliance with legal obligations is being questioned by forward-thinking hospital leaders, in recognition that more strategic resource allocation offers the potential to reduce financial risk associated with preventable emergency department and inpatient utilization. Recent actions in the policy arena to strengthen community benefit practices, as well as policies in related areas such as homelessness and behavioral health, challenge hospitals to strengthen their focus on prevention. At the same time, increased availability of data on health care costs, mapping of health care utilization patterns, and parallel overlays of hospital location, jurisdictional boundaries, and the social determinants of health offer significant potential for informed public dialogue at the regional level that builds an ethic of shared ownership for health across sectors. Local public health agencies can play an important role by establishing baselines, goals, and objectives in communities where health inequities are concentrated within county and municipal jurisdictional boundaries to align and focus the assets of health, community development, and business sector stakeholders.

## Introduction

The legal definition of charity for tax-exempt hospitals was expanded to the community benefit standard in 1969 with the issuance of IRS Revenue Ruling 69-545. The definition of charity moved beyond the “relief of poverty” interpretation to qualify hospitals that were “promoting the health of a class of persons that is broad enough to benefit the community”^1^. Impetus was provided in part by the prior passage of Medicare and Medicaid legislation, and an assumption that reduced demand for charity care would be insufficient to justify hospital tax-exemption.

The expanded standard has contributed to the development of a wide array of programs, services, and activities supported by hospitals across the country. Hospital engagement in these practices has become increasingly relevant in the context of the gradual and uneven, but inevitable movement toward risk-based reimbursement.

As providers and payers assume increasing financial risk for keeping people healthy and out of inpatient settings, they are coming to terms with the practical realities that there are factors that have a significant impact upon health and well-being at the individual, family, and community level. Awareness of the social determinants of health (SDoH) as an area of focus for community benefit expenditures is increasing, and while addressing these factors is outside of what is historically considered the responsibility of health care providers, the assumption of financial risk for their downstream impacts is bringing them into focus.

## A Brief History of Policies

Throughout the five-decade history of community benefit, the primary focus in the policy arena has been on the volume of hospital expenditures, and there has been ongoing debate about how much is enough and what kinds of expenditures are most appropriate.

Early challenges to the community benefit practices of tax-exempt hospitals came with class actions by municipalities in the northeastern U.S. in the late 70s and early 80s. Key drivers were increased pressure associated with the loss of tax revenue with outmigration of more affluent populations to the suburbs, and decreasing social safety net funding from federal and state governments. As urban tax-exempt hospitals served growing populations outside the geographic parameters of the municipality, city leaders began to question the deferral of their property taxes.

By the mid-1980s, states began to explore options for the development of community benefit statutes. There are a total of seven states which have established minimum financial thresholds for community benefit expenditures, the most recent addition of which is Oregon with HB 3076 (2019), and including Utah (1990), Texas (1993), Pennsylvania (1997), Illinois (2011), Nevada (2013), and Virginia (2013). While minimum thresholds ensure that hospitals meet a level of reported expenditures, they serve in practical terms as a “ceiling” rather than a “floor” for expenditures and may be a disincentive to focus on the content, geographic focus, quality and impacts of charitable services and activities. If that level is easily reached through documentation of spending on medical care for uninsured and underinsured populations, there may be less motivation to deploy resources for more proactive investments in prevention. That observation is supported by the research finding by Singh et al. (1) that minimum thresholds may result in an increased spending on direct patient care and lower levels of spending on community health improvement services. The same study also indicated that more comprehensive regulations (i.e., reporting requirements and at least one additional regulation) yielded higher overall spending.

Other states (e.g., NY in 1990, CA in 1994, NH in 2000) established what are referred to as “reporting laws,” which focus primarily on establishing a process for periodic assessment of community health needs, identification of priority content areas of focus, annual reporting on programs, services and activities, and establishment and description of institutional policies for financial assistance.

Community benefit standards received a major push at the federal policy level with the addition of the 501r elements of the Affordable Care Act, requiring community health needs assessments (CHNAs) and the development of formal implementation strategies, and revisions to the 990 Schedule H. The Schedule H revisions were driven by pressure from the Senate Finance Committee under the leadership of Charles Grassley, and reinforced by events in the field, not least of which were reports (2) of aggressive collection policies by Yale New Haven Hospital against patients who were subsequently judged to qualify for charitable support.

The revised Schedule H (form 990) includes a wide range of instructions^2^ and guidelines for CHNAs and Implementation Strategies, but the language in many cases is vague. For example, while hospitals are required to describe in their CHNA report “the evaluation of the impact of any actions that were taken,” (Part V, line 3i of Instructions), no further guidance is provided.

In another example, Section 501(r)(3) calls for hospitals to define their community of focus, taking into account “the geographic area served by the hospital,” “target populations served,” and “principal functions,” but cautions that “a hospital facility may not define its community in a way that excludes medically underserved, low-income, or minority populations who live in the geographic areas from which it draws its patients (unless such populations are not part of the hospital facility's target population or affected by its principal functions). “In a 2014, study conducted for the CDC (3) that reviewed CHNAs and Implementation Strategies in 15 regions, two health systems excluded proximal low income census tracts from their defined community benefit service area. In response to an inquiry as part of the study, they reported that their geographic parameters focused on their primary service area and they didn't judge the excluded census tracts as geographic areas from which they drew their patients.

HB 3076^3^ in Oregon represents a new level of oversight, one that offers both challenges and opportunities. It was passed in 2019 and will establish thresholds for individual or groups of hospitals and clinics within organizations to be reviewed and updated every 2 years. Criteria will include consideration of prior annual expenditures, community needs identified, workforce needs, financial status, demographics, spending on social determinants of health, taxes paid, public input, and reporting expectations for health professions education and research. This approach reflects an effort to accommodate the diversity in both hospital organizations and the communities they serve.

The language in HB 3076 gives attention to the SDoH as a priority, and explicitly includes “community building activities affecting health in the community” as a quantifiable community benefit [section 10 (2)(f)]. This is a subtle, but important move beyond the IRS 990 Schedule H requirements, which list community building categories in Part II of the reporting form as contributions not to be included in quantifiable totals. Hospitals are informed that “Some community building activities may also meet the definition of community benefit” and are instructed to document in Section VI “how the organization's community building activities, as reported in Part II, promote the health of the community or communities the organization serves.” Hospitals seeking to report community building activities as community benefits must then reclassify activities as community health improvement services. These instructions send a message that subcategories within community building are unlikely to be viewed by the IRS as legitimate, and hospitals must reclassify them as community health improvement services, even if the subcategories do not provide more appropriate options.

Legislative actions in areas outside of community benefit can play an important role in accelerating hospital collaboration with competitors to address the SDoH. In California, passage of Senate Bill 1152^4^ in July 2019 requires hospitals to establish a discharge planning policy and detailed written plan coordinating services, education and counseling and securing shelter for any patient for whom the absence of such services may result in negative health consequences. In a state with 26% of the homeless people in the U.S. (4), this new requirement has elevated the SDoH as an issue of immediate priority for both health care providers and payers there. A growing number of hospital collaboratives that have been formed there to co-invest in recuperative care centers, with active engagement and analysis of current social and related support service networks to better align and expand capacity.

Just as selected states have established minimum spending targets for primary care, some have suggested a need for similar thresholds for community health spending. Bakken and Kindig (5) offer projections to show that community health spending would increase three-fold if hospitals were required to spend 10% of community benefit dollars on community health improvement. Such an approach may address the concerns of some (6) that hospitals' interpretation of needs in CHNAs has the potential to medicalize poverty. A review of CHNAs will certainly include examples where stakeholders identify one or more SDoH as significant community needs, but a hospital may not select them as priorities based upon criteria that indicate a lack of expertise and experience within the hospital. That dynamic is shifting gradually as hospitals assume increasing financial risk for the downstream impacts of a lack of investment in the SDoH.

## Review of Practices

Hospital community benefit practices have undergone gradual change over the five decades of reporting, with examples of movement toward more evidence-informed interventions, increasing engagement of diverse community stakeholders to leverage internal resources, and the establishment of oversight structures. Most community benefit spending, however, involves a process of documenting the cost of providing services provided to uninsured, underinsured, and Medicaid patients, much of which involves high cost clinical services for preventable conditions.

In the 14 states that have not implemented Medicaid expansion, community benefit expenditures tend to be concentrated in the financial assistance reporting category. Predictably, for states that have implemented the Medicaid expansion, most of these expenditures shifted to Medicaid shortfalls. Among larger academic medical centers, net institutional costs associated with graduate medical education and research may represent the majority of community benefit expenditures^5^. Given the predominance of fee-for-service financing to date, there has been limited motivation for hospitals to move beyond a reactive approach to community benefit budgeting. One national study documented that only 5% of community benefit spending focuses outside of clinical settings, and only a small portion of that focuses on the SDoH (7).

The primary focus of research in the community benefit arena is on expenditures. For example, Singh et al. (8) found that overall spending is higher in counties with greater need, but there is not a corresponding increase in community health improvement services. This finding may reflect the practical reality that hospitals serving populations with greater needs (e.g., higher prevalence and acuity of chronic disease, behavioral health challenges), have lower margins due to higher percentages of Medicaid, and less discretionary dollars to spend on community health improvement services. Other hospitals have larger margins in part because their locations make them less likely to have low income people in their emergency departments. Without clear guidance and public expectations, hospitals located in more affluent communities are less likely to invest in prevention in communities not in their primary service area.

While there is limited evidence of a historical commitment by hospitals to address the SDoH (9), a recent study (10) documented 78 programs involving 57 health systems (representing 917 hospitals) with $2.5 billion in health system funds allocated, including $1.6B in housing interventions. There is growing evidence that federal agencies are interested in encouraging these kinds of resource allocations, reflected most recently in the public statements of Alex Azar, HHS Secretary (11).

Investment in research on health outcomes associated with community benefit expenditures has been constrained by a regulatory focus on the volume of expenditures. The institutional focus on compliance with documenting expenditures related to deferred tax revenue creates a disincentive for investment of hospital resources to evaluate impacts, to align assets across competitive and sectoral lines to scale efforts, and to geographically focus assets where health inequities are concentrated.

There are, however, increasing opportunities to document reduced costs associated with preventable utilization in emergency department and inpatient settings. A 2013–2014 retrospective cohort study of community benefit spending showed that hospitals with the largest percentage of spending in community social needs had substantially lower readmission rates (12). A recent review of studies of expenditures on SDoH found 12 of 39 studies focused on housing, and 10 of those 12 documented improvements to health outcomes and/or reduced costs (13). Expansion of risk-based reimbursement, growing knowledge of the impact of the SDoH, increased transparency in health care costs, and attention to geographic patterns in service utilization are all key levers with the potential to change these historical patterns.

## Looking Ahead: The Next Half Century

Public scrutiny into the charitable practices of non-profit hospitals is on the rise again, with cities in states such as New Jersey (14) and Pennsylvania (15) threatening to end tax deferments. One factor is evidence of high profitability among selected hospitals. A recent commentary (16) indicated that seven of the 10 most profitable hospitals in the U.S. are non-profits and since the passage of the ACA, revenue in more profitable hospitals has increased 15% while their charity care numbers dropped 35%. Though overall profitability among non-profit hospitals is low, reports of these outliers contribute to a negative public perception. Recent studies also suggest that hospitals in Medicaid expansion states provided less total charity care (i.e., financial assistance and Medicaid shortfalls) relative to net operating revenue (17), and that differences in non-operating income do not influence total spending on community benefit (18).

Growth in risk-based reimbursement presents significant challenges to providers to integrate clinical care management strategies with social services *and* community level interventions that address the SDoH. Challenges documented in a recent study of Accountable Care Organizations (19) include; (a) short funding cycles and different time horizons for return on investment, (b) limited knowledge of social service organizational capacity, (c) inadequate data on patient social needs, and (d) undeveloped local/regional partnerships. Recommended actions include policies to provide sustained funding to support deeper working relationships and data standardization. Even if strong partnerships and data systems are established, there is emerging evidence of diminishing returns from interventions that only address clinical and social service needs at the level of the individual patient (20).

Increased transparency (e.g., cost of services, use of GIS technology, data sharing across sectors), increasing timely access to quality primary care, and recognition of the importance of addressing the SDoH in a risk-based financing environment offer considerable potential to strengthen community benefit practices. Key actions moving forward include:

- Establish uniform criteria that clarify which services/activities qualify as community benefits, including a requirement for a primary focus in sub-geographic areas where health inequities are concentrated.
- Provide funding and related incentives for alignment of services/activities and ongoing monitoring and evaluation at the regional level across organizations and sectors.
- Give attention to comparative analysis of community benefit expenditures at the regional level related to facility proximity to low income communities, jurisdictional boundaries, and hospital payer mix.

There is growing evidence that non-profit hospitals are gaining knowledge and awareness of the important potential role they can play as partners, not just in providing high quality acute care, but in improving health and well-being in local communities. While public policy also has a role to play, much can be accomplished through strategic use of information technology and generative dialogue among community and institutional leaders in multiple sectors in communities across the nation. With the appropriate funding and collaboration with public and private sector partners, local public health agencies are well-positioned to support planning, design, and monitoring of more strategically aligned and focused resource allocations by hospitals and community partners. As the field of community benefit enters its second half century, hospitals leaders will be increasingly challenged to work across sectors and to share ownership for reducing costs and improving health in our communities.

## Data Availability Statement

The original contributions presented in the study are included in the article/supplementary material, further inquiries can be directed to the corresponding author.

## Author Contributions

KB conceived this article, wrote, and revised it.

## Conflict of Interest

The author declares that the research was conducted in the absence of any commercial or financial relationships that could be construed as a potential conflict of interest.

## Footnotes

^1^Rev. Rul. 69-545, 1969-2 C.B. 117, paragraph 13.

^2^https://www.irs.gov/pub/irs-pdf/i990sh.pdf

^3^https://legiscan.com/OR/text/HB3076/2019

^4^https://leginfo.legislature.ca.gov/faces/billNavClient.xhtml?bill_id=201720180SB1152

^5^For example, in 2017, The Johns Hopkins Hospital reported a total of $267M of community benefit expenditures, $187M of which was for health professions education (HPE) shortfalls; the University of Chicago Hospital reported a total of $194M, of which $70M was for HPE and $48M for research; Georgetown University Medical Center reported $63.7M, of which $49M was for HPE, Emory University Hospital reported $567M, $264 of which was for HPE and $118.7M in research, and University of Maryland Medical Center reported $192M, of which $166.7 was for HPE – Data source: Community Benefit Insight (http://www.communitybenefitinsight.org/)
